# The Habitual Diet and Avocado Trial (HAT): A Collaborative Multicenter Model for Large-Scale, Privately Funded Nutrition Research Consortia

**DOI:** 10.1016/j.cdnut.2026.107713

**Published:** 2026-05-13

**Authors:** Penny M Kris-Etherton, Zhaoping Li, Nirupa R Matthan, Sujatha Rajaram, Joan Sabaté, David M Reboussin, Nikki Ford, Samuel Barnes, Kristina S Petersen

**Affiliations:** 1 Department of Nutritional Sciences, The Pennsylvania State University, University Park, PA, United States; 2 Center for Human Nutrition, David Geffen School of Medicine at UCLA, Los Angeles, CA, United States; 3 Jean Mayer USDA Human Nutrition Research Center on Aging, Tufts University, Boston, MA, United States; 4 Center for Nutrition, Healthy Lifestyle and Disease Prevention, School of Public Health, Loma Linda University, Loma Linda, CA, United States; 5 Department of Biostatistics and Data Science, Wake Forest University School of Medicine, Winston-Salem, NC, United States; 6 Avocado Nutrition Center, Mission Viejo, CA, United States; 7 Department of Radiology, School of Medicine, Loma Linda University, Loma Linda, CA, United States

**Keywords:** Habitual Diet and Avocado Trial, research model, multicenter clinical nutrition research study, visceral adiposity, avocado, clinical trial

## Abstract

The research model used to conduct the Habitual Diet and Avocado Trial (HAT), a large multicenter clinical trial (*n* = 1008 participants) designed to evaluate the effect of consuming 1 avocado/d for 6 mo on visceral adiposity in individuals with an increased waist circumference, has been a long-term productive collaboration. The primary outcome analysis showed that intake of 1 avocado/d did not affect visceral adiposity compared with habitual dietary intake. Secondary and ancillary analyses showed improvements in diet quality, LDL cholesterol, and red blood cell fatty acid composition, as well as the gut microbiome with avocado intake. These findings are relevant to a large proportion of the United States population because of the study sample characteristics. The aim of this paper is to summarize the research protocol and implementation of the HAT as well as the findings. This approach could be used by consortia supported by nongovernmental entities. Notably, there is still ongoing research being conducted by the investigators using the data collected from the HAT. Moreover, HAT samples and data are available, pending an approved request, to scientists interested in conducting avocado health research. The collaborative commitment of the investigators, their collegial spirit, along with strong leadership and ongoing support from the coordinating center and funding agency representatives, has created a productive research consortium. This consortium has furthered knowledge about the health effects of avocados, and the approach has maximized the return on the research investment from a single large clinical trial.

## Introduction

Many clinical trials examining the health effects of single foods have been conducted, and the findings have informed recommendations for healthy eating/dietary patterns. These trials are typically limited in scope (and outreach) for various reasons, including the funding limits, timeline, and research priorities set by the sponsor, which are driven by the short and long-term strategic goals of the funding organization. This results in a focused trial with a limited scope. Typically, a primary endpoint paper is published (that includes the prespecified primary and secondary outcomes), and sometimes other endpoints (exploratory) are assessed and published, which may occur sometime later. Although prevalent and useful in advancing the field, small single-center clinical trials typically yield less research output and fewer publications over time than larger multicenter trials. Multicenter trials typically evaluate more participants and, consequently, include participants who are more representative of the United States demographics, which makes the results more generalizable to public health policy.

Typically, funding for research on single foods is obtained after approval of an investigator-initiated proposal submitted to a commodity/private sector group. In some cases, funding entities will award several grants in response to a request for proposals that address multiple different focus areas or multiple awards focused on a particular area. Herein, we discuss the implementation of an alternate private sector-initiated multicenter trial approach that was used for the Habitual Diet and Avocado Trial (HAT). The HAT was a multicenter trial with 4 clinical centers and a coordinating center designed to evaluate the effect of consuming 1 avocado per day on visceral adiposity (primary endpoint) and other cardiometabolic risk factors (secondary endpoints) in 1008 participants [1,2]. To further evaluate the health effects of consuming 1 avocado/d, numerous ancillary studies were conducted. The findings from this trial have contributed to our understanding of the health effects of avocados and will continue to do so because of the established strong consortium and the infrastructure utilized to promote data integrity and data sharing.

## Background

HAT, a partnership between private sector and university scientists, was conducted between 27 June, 2018 and October 2020, with the primary endpoint paper published in 2022 [2]. Hass Avocado Board’s Avocado Nutrition Center (sponsor) identified a need for a large multicenter study that included participants generally representative of the United States population across gender, age, racial, and ethnic groups to examine the health effects of avocados under real-world conditions. The sponsor engaged a directed approach to recruit collaborative-focused and recognized scientists with the requisite expertise and geographical representation to conduct this type of food-based, multicenter clinical nutrition research, and a consortium was formed. A Steering Committee, comprising ≥1 (often 2+) academic research representatives from each clinical site, the coordinating center, and 1 sponsor (from Hass), designed and conducted the trial. Decisions were made by majority vote, ensuring that the sponsor held only a single vote, whereas the academic sites comprised the voting majority. All research was further conducted in accordance with the American Society for Nutrition Guiding Principles and the Hass Avocado Board Guiding Principles [3,4]. The trial was registered (NCT03528031), and a centralized Institutional Review Board (IRB) was used. All the data were uploaded to a web-based system developed by the coordinating center. The sponsor did not have access to the data nor participate in data interpretation or manuscript preparation, but, much like a project officer for an NIH U Grant Consortium Agreement, played a key role in facilitating and monitoring study activities (in collaboration with the coordinating center). This may be an approach that other funding entities could adopt to provide a rigorous, externally valid evaluation of the health effects of a food/product of interest.

The scale of HAT provided the opportunity to conduct many ancillary studies designed to evaluate the effects of avocado consumption on cardiometabolic health in different populations, their accompanying metabolic and clinical effects, and their effect on diet quality. Some of the ancillary studies were planned as the primary endpoint protocol was being created by the investigators (described in the HAT design manuscript [1]), whereas some were proposed both during and after the HAT (Table 1 [[5], [6], [7], [8], [9], [10], [11], [12], [13], [14]] describes the studies that have been conducted and are ongoing).

**TABLE 1:** HAT ancillary studies—completed

Lead clinical center	Aim	Major finding(s)
A. Effects of avocado consumption on diet quality		
Penn State	Examine changes in the HEI-2015 after a food-based intervention and assess the associations between HEI-2015 change and intervention effects on cardiometabolic risk–related outcomes [5].	The AD group had a greater increase in the HEI-2015 (4.74 points) than the HD group. The AD group had greater increases in the following HEI-2015 components: total vegetables (0.99 points), fatty acid ratio (2.25 points), sodium (1.03 points), refined grains (0.82 points), and added sugars (0.84 points).
Loma Linda	Assess the effect of consuming a large avocado (168 g, 281 kcal) daily in the AD group compared with the HD group on food and nutrients [6].	The AD had a higher daily intake of energy, potassium, fiber, and a lower daily intake of animal protein compared with the HD group that abstained from eating avocados. Food group analysis revealed a lower consumption of animal-derived protein from red meat, processed meats, poultry, and fish in the AD group, compared with the HD group.
Loma Linda	To examine the effect of including 1 avocado daily in the AD group compared with the HD group on the glycemic index and glycemic load in free-living adults with overweight/obesity [13].	Daily consumption of 1 avocado within the AD significantly reduced glycemic load, but not glycemic index, without requiring significant dietary changes.
B. Health effects of avocado consumption		
Penn State	To evaluate the effect of providing 1 avocado/d for consumption over a 6-mo period on central blood pressure, augmentation index, pulse wave velocity, and flow-mediated dilation, compared with the HD control group [7].	There was no effect of avocado consumption on measures of vascular function.
Penn State	Examine the effect of daily avocado intake for 26 wk on Life’s Essential 8 in adults with abdominal obesity [8].	Intake of 1 avocado/d for 26 wk did not significantly affect the total cardiovascular health score in United States adults with abdominal obesity. Diet quality, sleep health, and blood lipids, however, all improved with daily avocado intake.
Tufts	Effect of daily avocado consumption for 6 mo compared with HD on red blood cell fatty acid profiles and association with cardiometabolic risk factors in individuals with abdominal obesity: a randomized trial [9].	Daily avocado consumption over 6 mo significantly increased MUFA 18:1n−7c in RBCs and potentially attenuated some of the unfavorable individual RBC fatty acid–cardiometabolic risk factor associations observed over time in the HD group.
Tufts	Serum metabolite profiles in adults with abdominal obesity in response to consuming 1 avocado daily for 6 mo: an exploratory analysis [10].	Avocado intake was associated with subtle shifts in serum metabolites related to lipid, carbohydrate, and amino acid metabolism, with weak effects on visceral adipose tissue volume, plasma triglyceride, and total cholesterol concentrations.
UCLA	To evaluate the impact of daily avocado consumption on gut microbiota in adults with abdominal obesity [11].	Avocado consumption increased microbial diversity, with the most pronounced effects observed in participants with poor baseline HEI-2015 scores. Long-term avocado intake (26 wk) had a greater impact on microbiome composition compared with short-term intake (4 wk).
UCLA	To evaluate biomarkers related to metabolic dysfunction-associated steatotic liver disease using contrastive learning, a multimodal representation learning approach, from the MRI liver scans of HAT participants.	The artificial imaging-guided immunosensor derived from a metal-organic framework quantified 6 plasma biomarkers, aspartate aminotransferase, alanine aminotransferase, γ-glutamyl transferase, cathepsin D, cytokeratin-18, and fibroblast growth factor 21 that were associated with metabolic dysfunction-associated steatotic liver disease. Manuscript under review.
Loma Linda	Effects of 1 avocado/d for 6 mo on cognitive performance in overweight adults: a randomized controlled trial [14].	The consumption of 1 avocado/d without any additional lifestyle modifications for 6 mo did not significantly alter cognitive function in adults with central obesity.
C. Adherence and subjective outcomes		
UCLA	Examine the adherence, changes in weight, and waist circumference associated with the daily consumption of a culturally preferred food, namely an avocado, among Hispanic/Latina females in HAT [12].	The Latina adult females showed high adherence to the 6-mo dietary intervention when avocados—a culturally preferred food—were included. Providing avocados likely improved compliance by reducing financial barriers and addressing food insecurity, a common challenge in Latin communities. Despite increased avocado intake, there were no significant differences in weight or waist circumference between groups over 6 mo.
Hass Avocado Board, Coordinating Center & Tufts	Evaluate the effect of daily avocado consumption on health-related quality of life.	Eating 1 avocado daily for 6 mo did not improve health-related quality of life domain or composite scores compared with HD. Satisfaction with the avocado intervention was positively associated with mental well-being. Manuscript under review.

Abbreviations: AD, avocado-supplemented diet; HAT, Habitual Diet and Avocado Trial; HEI, Healthy Eating Index; HD, habitual diet; RBC, red blood cell; UCLA, University of California, Los Angeles.

## The HAT Research Consortium Activities

**FIGURE 1:**
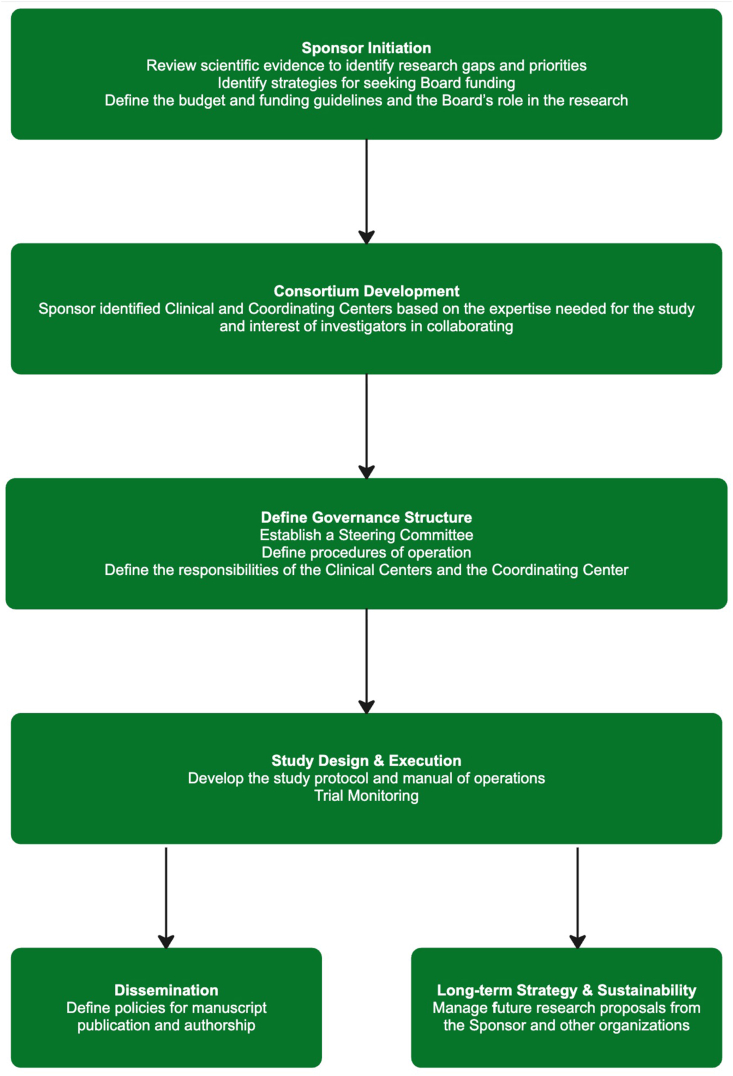
Overview of the HAT research consortium activities. HAT, Habitual Diet and Avocado Trial.

Detailed guidelines for designing, conducting, documenting, and reporting human nutrition randomized controlled trials (including multicenter studies) for achieving high scientific rigor have been reported [[15], [16], [17], [18], [19], [20]]. Briefly, the key strengths of a multicenter study are the ability to recruit larger numbers more rapidly, the greater generalizability of the cohort, and the opportunity to involve a larger group of collaborators. These principles were central to designing HAT. In addition, we followed the updated framework for industry funding of food and nutrition research [21]. Beyond the design of HAT, we report a research model that has been very productive and maximized the data collected in the main clinical trial. The HAT research model is described below and summarized in Figure 1.

### Sponsor initiation: review scientific evidence, identify research gaps, and define funding strategies, budget guidelines, and the board’s role

Research proposed is typically justified on the basis of gaps in the scientific evidence base. Beyond identifying research needs in nutrition (based on a literature review, unresolved scientific questions, and pressing health-related issues), the next step is to find research funding. For commodity organizations and private sector groups, the lead scientist(s) from the organization must champion the effort; they are best positioned to do this, being well aware of the board’s interest and funding availability. It is important to determine how much funding is available for the project, what the schedule is for funding, and what the funding guidelines are (i.e., expenditures allowed). During the implementation of the research and after the research is done, the lead scientist(s) provide project updates to the board.

### Consortium development: identify clinical and coordinating centers based on expertise and interest in collaboration

The sponsor identified and approached a scientist(s) from each clinical center and the coordinating center. Implicit to the need for the investigators to have the requisite expertise, facilities, and equipment to conduct the study, they must be willing to commit to a long-term collaboration (to complete ongoing ancillary studies, report on them at scientific meetings, and prepare new grants). HAT investigators had unique expertise that collectively added to the science that could be done, including providing many training opportunities for junior investigators and students. Penn State, Tufts, and Loma Linda Universities shared expertise in diet assessment, which included assessing diet quality and glycemic index/load, as well as assessing risk factors for cardiometabolic diseases. Penn State also had expertise in vascular health, and Loma Linda had expertise in assessing cognition. Tufts University had expertise in fatty acid metabolism, cholesterol synthesis, and metabolomics, and University of California, Los Angeles had expertise in implementing weight management programs and assessing the microbiome, genetic variants, and artificial intelligence modeling. Collectively, the individual and shared expertise of the clinical centers was instrumental in generating meaningful scientific insights into the health effects of avocado consumption. The geographical dispersion of clinical centers facilitated studying a diverse participant population. The coordinating center at the Wake Forest University School of Medicine was selected based on its extensive experience with multicenter studies.

### Define the governance structure: establish a steering committee, define the procedures for operation and responsibilities of the clinical centers and the coordinating center

The HAT Steering Committee included all clinical center and coordinating center investigators, along with 1 representative from the Hass Avocado Board. Although a large group, this membership allowed all to contribute to HAT. This inclusive approach created an environment that elicited many research ideas and protocols for ancillary studies. The Steering Committee always welcomed ideas for proposed ancillary studies that were reviewed for approval. In addition, there was an inclusive authorship policy on all papers for which investigators contributed. The Steering Committee met regularly with the coordinating center, proposing a schedule and agenda for all meetings.

The main responsibility of the HAT clinical centers was to conduct the studies proposed according to the approved protocol(s). The coordinating center served as the main hub to oversee all HAT research activities. Additional coordinating center responsibilities, in collaboration with the clinical centers, were to develop the clinical trial protocol for HAT, write a protocol manual (including data collection tools and other systems), lead regulatory compliance activities, and submit a common IRB proposal for the main study. The individual clinical centers submitted IRB proposals for the ancillary studies at their respective universities. Any consortium scientist who wished could be a collaborator. The progress of these studies was monitored during the monthly Steering Committee calls until the paper was accepted for publication. Major responsibilities of the HAT coordinating center were to support the design of the trial, manage, monitor, and analyze the data that were generated by the clinical centers, and to provide administrative support. The coordinating center created a HAT website that was used for communication purposes while the clinical trial was ongoing.

### Study design and execution: development of the study protocol and manual of operations, and trial monitoring

The HAT Steering Committee met once monthly to discuss aspects of the study, including research papers, ancillary studies, and scientific presentations. Importantly, the HAT Steering Committee designed the primary research study that was conducted, as well as defined the clinical trial’s primary endpoint, and prioritized secondary endpoints based on scientific importance and budget considerations. In addition to monthly HAT Meetings, regular research meetings were held to discuss all aspects of the ongoing HAT, as well as the ancillary studies that were proposed or ongoing. These meetings were held every other week during trial start-up through completion to troubleshoot issues and update protocol procedures/materials. The coordinating center led these meetings; representatives from the Hass Avocado Board did not attend.

### Dissemination: define policies for manuscript publication and authorship

The Steering Committee developed a policy for manuscript publication and authorship. For the protocol and the primary publication, all Steering Committee members were involved in the manuscript preparation, and the prominent authorship positions were for members who led the manuscript preparation. For ancillary projects, plans for publication were included as part of the ancillary project proposal. Writing groups open to any consortium member were established for these papers.

### Long-term strategy and sustainability: manage future research proposals submitted by HAT investigators and other scientists, and share HAT data and samples with the scientific community

**FIGURE 2:**
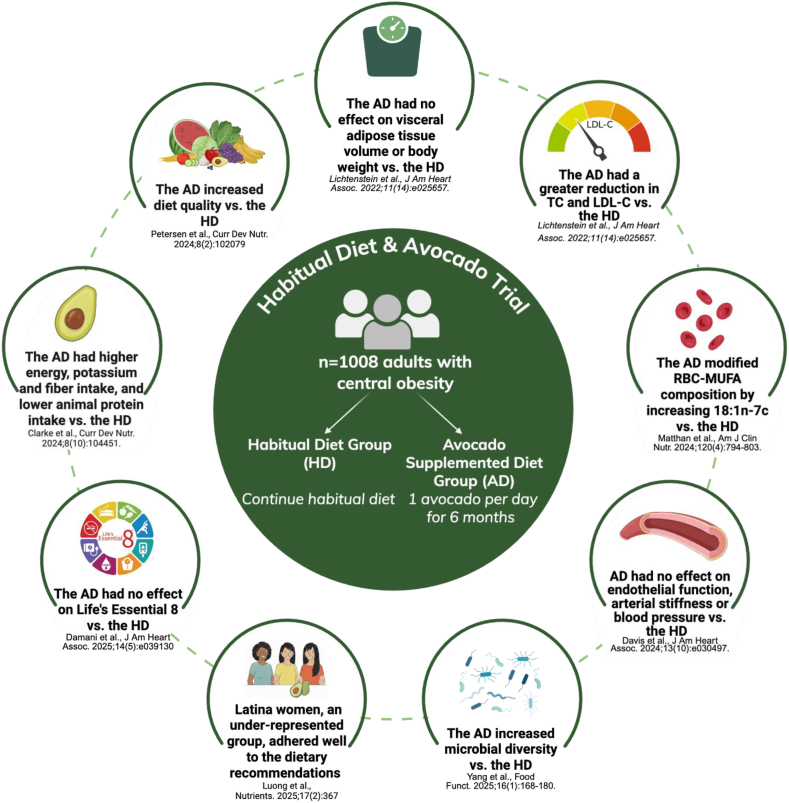
Overview of the HAT design and key findings. AD, avocado-supplemented diet; HAT, Habitual Diet and Avocado Trial; HD, habitual diet.

At any point, an investigator involved in the consortium could propose an ancillary study [1]. The review process involved preparing a short proposal with the rationale, aims, and methodology that was sent to the Steering Committee. At the Steering Committee meeting, these were discussed and voted on. Many ancillary studies have been completed [[5], [6], [7], [8], [9], [10], [11], [12], [13]] as summarized in Table 1, and several are ongoing. Collectively, the ancillary studies have leveraged the available dataset to provide further evidence on the diet and health effects of avocados (Figure 2).

## HAT Collaborative Opportunities

There is also an opportunity for scientists to conduct secondary data analyses with our data and data from other studies funded by the Hass Avocado Board (details can be found at: https://research.loveonetoday.com/research-opportunities/). Table 2 shows the schedule of measurements for HAT. MRIs were conducted to evaluate the primary endpoint measurement, visceral adiposity, as well as hepatic fat. Standard biochemical assays were done on serum/plasma and red blood cells, as well as on feces, the latter to assess gut microbiota. Questionnaires were also used to assess dietary intake, diet quality, diet satisfaction, and sleep quality. Biological samples are also banked. Both data and biological samples are stored according to the best practices for clinical studies [18]. Thus, there are many opportunities for follow-up collaborative research. Interested investigators may inquire through the sponsor website (https://research.loveonetoday.com/research-opportunities/), whereby a proposal will be requested for Steering Committee review and approval.

**TABLE 2:** Schedule for sample and data collection^1^

Measurement	Visit 1 and 2 (–2 to 0 wk)	Visit 3 (4 wk)	Visit 4 (8 wk)	Visit 5 (12 wk)	Visit 6 (16 wk)	Visit 7 (20 wk)	Visit 8 (26 wk)
Screening interview	x						
Health and demographics	x						x
Blood pressure	x	x	x	x	x	x	x
Height	x						
Weight	x			x			x
Waist circumference	x			x			x
Visceral adiposity, MRI	x						x
Hepatic fat, MRI	x						x
Triglycerides	x			x			x
Cholesterol	x			x			x
Fasting glucose	x			x			x
Fasting insulin	x			x			x
hsCRP	x			x			x
RBC fatty acid profile	x			x			x
Buffy coat DNA	x						
Serum, plasma and RBC	x			x			x
Gut microbiota	x	x					x
24 h diet recall	x		x		x		x
Diet and avocado satisfaction	x			x			x
Quality of life (Short Form-36)	x			x			x
Quality of life (Short Form-20)		x	x		x	x	
Sleep quality	x			x			x
Cognition assessment	x						x

Abbreviation: hsCRP, high sensitivity C reactive protein; RBC, red blood cells.

1 Adapted from Reboussin et al. [1].

## Study Challenges

HAT used a rolling enrollment design. The first participant began the trial in June 2018, and the last participant started in March 2020. With this research design, the workflow was manageable, and as specific questions arose about participants (eligibility, special needs, etc.) and the methods (implementation, etc.), the Steering Committee was set up to respond to queries in a timely and consistent manner. Having the Steering Committee’s role well defined beforehand and a collegial group of investigators helped resolve challenges so the research could continue on schedule. As would be expected in any clinical trial, there were challenges that arose that warranted quick attention. A few examples are described.

### Unexpected surge in public interest in HAT following media coverage

During the early recruitment efforts, the national media learned about the study and published a news article with recruitment information. The clinical sites were inundated with calls from interested potential participants from across the United States that overwhelmed one of the study center’s websites for 48 h. To address this “explosive enthusiasm” for the study, much staff time and the intervention of the information technology team at the university were required.

### Standardizing MRI scan measurements at the clinical sites

To ensure data were consistent across MRI scan measurements performed by 7 different scanners at the 4 clinical centers, a traveling phantom was built and traveled twice to all imaging sites. The phantom was scanned at all sites before any participants were enrolled to ensure the scan protocol was properly configured and that the scans produced consistent values. After ∼6 mo, the phantom was scanned again to ensure stable values over time. Additional details on phantom construction and imaging protocol have been previously published [22].

The traveling phantom showed consistent values across scanners and over time. No significant differences were detected between the 7 MRI scanners or between the first and second measurements. The SD for fat volume measurements was 14.l mL on a total phantom fat volume of 552 mL, or 2.6% of the measured fat volume.

### Navigating the COVID-19 pandemic quarantine

The biggest challenge HAT had to address was the lockdown due to the COVID-19 pandemic. Before the clinical research was paused, 792 participants had a follow-up MRI scan (for the primary study endpoint) and had essentially completed the HAT. Because of the COVID-19 research pause, the remaining HAT participants (*n* = 216) continued their participation in the study beyond the 6-mo intervention period virtually (i.e., those in the avocado group had avocados delivered to their homes so they could continue incorporating 1 avocado/d in their diet; the habitual diet group continued after their usual dietary pattern). Each clinical center was allowed to resume clinical research activities at different times, taking extraordinary precautions to ensure participant safety. There were many challenges during this period of time, including encouraging participants in the avocado group to continue eating 1 avocado/d until they had their final MRI done (and also encouraging the control group participants to continue participation). Other challenges were submitting a detailed proposal to the IRB for keeping participants safe during the final stages of the intervention, which increased staff workload appreciably. Despite these challenges, the study completion rate was 93%.

## Discussion

Clinical nutrition research has evolved over the years from the conduct of relatively small studies designed to address nutrient deficiency and therapeutic nutrition topics to currently addressing larger-scale global health issues that are impacted by dietary patterns and food systems [23]. It is well recognized that advancing nutrition science and promoting optimal nutrition universally requires extensive and rigorous scientific investigation to identify healthy dietary patterns for different individuals and populations for health and wellness. Ongoing investigations in many areas, including the microbiome, metabolomics, and personalized nutrition, are seeking to achieve global health goals through better nutrition practices. Given the need to address complex nutrition problems comprehensively and quickly, new models for clinical nutrition research are needed. Larger multicenter studies can be conducted to advance science relatively expeditiously because of their large sample size and associated ancillary studies. We described a research model that reinforces the value of conducting a large multicenter study, along with many ancillary studies that increased our understanding of the health effects of avocados. We believe this model could be effective (and efficient) in addressing research questions related to the universal goal of achieving a healthy dietary pattern with specific food recommendations for all individuals and populations.

We would be remiss not to acknowledge federally funded multicenter studies that evaluated the health effects of dietary patterns, most notably the Dietary Approaches to Stop Hypertension (DASH) [24], as well as the Mediterranean-DASH Intervention for Neurodegenerative Delay [25] trials and also specific nutrients (i.e., different types and amounts of fatty acids) as was done in the Dietary Effects on Lipoproteins and Thrombogenic Activity (DELTA) studies [26,27]. In addition, another seminal study conducted in Spain, the Prevención con Dieta Mediterránea Trial, evaluated the cardiovascular effects of a Mediterranean dietary pattern with food supplements (i.e., extravirgin olive oil and nuts—walnuts, almonds, and hazelnuts) [28]. The National Heart, Lung, and Blood Institute sponsored Biologic Specimen and Data Repository Information Coordinating Center (BioLINCC) websites for the DASH Study (https://biolincc.nhlbi.nih.gov/studies/dash/) and the DELTA Study (https://biolincc.nhlbi.nih.gov/studies/delta/) provide considerable information about the research that was conducted, including the study protocols and key publications. The HAT research program implemented the rigor of these landmark trials, and also was uniquely different in that it focused on a single food, and research continued by all investigators for years after completion of the clinical trial. Overwhelmingly, the key uniqueness of the HAT research has been the ongoing commitment of the study researchers. Having ended the clinical trial over 3 y ago, the ongoing interactions of the researchers importantly contributed to the sustained productivity of the team. In addition, other distinguishing characteristics of HAT include the diversity of the study population, rigorous methods of measurement of adiposity, and the breadth of ancillary study research.

In summary, we describe a research model that we used to conduct single food-based research that received private sector funding. Importantly, the research process followed the updated framework for industry funding of food and nutrition research [21]. The multicenter research model we implemented was productive, providing an opportunity to evaluate a large sample size (>1000 participants) and creating many opportunities for the conduct of ancillary studies that advanced our understanding of the multiple health effects of avocados.

## Author contributions

The authors’ responsibilities were as follows – PMK-E, ZL, NRM, SR, JS, DMR, NF, KSP: conceptualized this manuscript; KSP: generated the figures; PMK-E, KSP: had primary responsibility for the final content; and all authors: contributed to drafting and editing the manuscript, read and approved the final version.

## Data availability

Investigators interested in accessing the HAT data (and banked samples) may inquire through the sponsor website (https://research.loveonetoday.com/research-opportunities/).

## Declaration of Generative AI and AI-Assisted Technologies in the Writing Process

The authors declare that no generative AI or AI-assisted technologies were used in the writing of this manuscript. Google Gemini was used in the preparation of the figures.

## Funding

This work was supported by the Avocado Nutrition Center.

## Conflict of interest

DMR reports financial support was provided by Hass Avocado Board Avocado Nutrition Center. NF is an employee of the Hass Avocado Board. The other authors declare that they have no known competing financial interests or personal relationships that could have appeared to influence the work reported in this paper.
